# Could we accidentally measure visual acuity low in the time of the COVID-19 pandemic?

**DOI:** 10.5935/0004-2749.20200110

**Published:** 2024-02-11

**Authors:** Remzi Karadag

**Affiliations:** 1 Veni Vidi Eye Center, Caddebostan, Kadikoy, Istanbul, Turkey; 2 RK Eye, Aesthetics and Health Services, Kadikoy, Istanbul, Turkey

Coronavirus-19 disease (COVID-19) is a life-threatening condition caused by severe acute respiratory distress syndrome coronavirus-2 (SARS-CoV-2). Respira tory droplets generated by infected individuals and direct contact with virus-contaminated fomites are thought to be the main routes of transmission for the human coronavirus^(1)^. Because COVID-19 is transmitted by droplets and close contact, and because ophthalmologists must be positioned close to their patients during an examination, they are at increased risk for this infection. Therefore, patients, technicians, and physicians should use personal protective equipment during an examination. Protective glasses, face mask/shield, and shield attached to a biomicroscope should be used to prevent transmission by ophthalmologists. In addition, patients should at least wear a mask covering the mouth and nose^(2^-^4)^.

Wearing a mask has become a part of life during the COVID-19 pandemic. Young et al. reported that poorly fitting face masks represent a new cause of visual field artifact that can mimic pathological field defects in a patient. They stated that adjusting the fit of face masks may help to prevent fogging or mask slippage and increase test reliability^(5)^. Medical staff members may complain about glasses being fogged when caring for COVID-19 patients. Fogging can affect visual acuity, thereby preventing nurses’ work efficiency. This can cause improper blood collection, delay tracheal intubation and deep vein catheterization, or even result in injury to patients^(6)^. Similarly, because patients must continue to wear their masks during the refraction examination, fogging may occur on the trial glasses, and this can affect their visual acuity. During a trial of glasses and frames, the doctor can easily notice any fogging effect, and because the trial glasses are removed when the glasses are replaced, this will not have much of an effect on patient vision.

With regard to the phoropter device, refraction glasses are located inside the device, and there are fixed glasses (oculars) in front of and behind them. Therefore, the oculars of the phoropter may become fogged and affect the patient’s vision. This can be easily noticed only in patients who have refractive errors. During the examinations of patients with eye diseases such as retinal disorders, cataracts, glaucoma, corneal disorders, and uveitis, fogging might occur on the glasses, and if this situation is not noticed, the vision level of the patient may be mistakenly evaluated as low, which can affect patient treatment. Ophthalmologists should keep in mind that an unexpected reduction in visual acuity of patients wearing a mask may be the result of fogging. In addition, fogging can become worse cold weather. If the visual acuity examination is performed by an ophthalmologist, he or she may notice an unexpected decrease in visual acuity due to fogging. However, technicians, nurses, or students examining patient visual acuity may not notice this phenomenon. Some commonly used methods to prevent fogging including using washing-up liquid or hand sanitizer and applying antifogging agents or iodophors^(7^-^10)^. In addition, the top portion of the patient’s mask may be fixated with a skin plaster, and the oculars of the phoropter can be wiped with anti-fog solutions to avoid fogging of the oculars of the phoropter. In addition, during refraction examination, the patient can be provided with a mask to cover only his or her mouth and leave the nose open. If, despite all precautions, fogging still occurs, the fog should be removed occasionally with a suitable cloth.
